# Proximity extension assay revealed novel inflammatory biomarkers for follicular development and ovarian function: a prospective controlled study combining serum and follicular fluid

**DOI:** 10.3389/fendo.2025.1525392

**Published:** 2025-02-10

**Authors:** Chong Wang, Ying Feng, Yu Chen, Xianhua Lin, Xiangjuan Li

**Affiliations:** ^1^ Department of Reproductive Medicine, Hangzhou Women’s Hospital, Hangzhou, China; ^2^ Department of Clinical Laboratory, Hangzhou Women’s Hospital, Hangzhou, China; ^3^ Department of Obstetrics and Gynaecology, Hangzhou Women’s Hospital, Hangzhou, China

**Keywords:** follicular development, ovarian function, proximity extension assay, follicular fluid, serum

## Abstract

**Background:**

Many components in follicular fluid (FF), such as peptide hormones, cytokines, and steroids, undergo dynamic changes during folliculogenesis and have important roles in follicular development. Because systemic inflammation has also been found to contribute to diminished ovarian reserve (DOR) in previous studies, do certain serum/FF inflammatory biomarkers affect both follicular development and ovarian function?

**Methods:**

Serum samples from the menstruation phase (n=26), serum samples from the ovulation phase (n=26), FF samples of mature oocytes (n=26), and FF samples of immature oocytes (n=10) were collected. Olink proteomic proximity extension assay (PEA) technology was used to compare the differentially expressed proteins (DEPs), and patients were divided into two subgroups—the normal ovarian reserve (NOR) group and the DOR group—for further bioinformatics analysis and verification by enzyme-linked immunosorbent assay (ELISA).

**Results:**

In total, 16 DEPs were detected between the mature group and the immature group (FF), and 11 DEPs were detected between the ovulation group and the menstruation group (serum). Further subdivision of the ovarian reserve subgroups revealed 22 DEPs in FF and 3 DEPs in serum. Among all four comparisons, only the expression of oncostatin M (OSM) significantly differed. The OSM signaling pathway, the IL-10 anti-inflammatory signaling pathway, and the PI3K−Akt signaling pathway are three notable pathways involved in affecting ovarian reserve capacity according to bioinformatics analysis. In addition, the concentration of estradiol on the hCG day was slightly but positively correlated with OSM (*r*=0.457, *P*=0.029). A significantly greater level of OSM (5.41 ± 2.65 vs. 3.94 ± 1.23 pg/mL, *P*=0.007) was detected in the serum of NOR patients via ELISA verification, and the sensitivity and specificity of ovarian reserve division were 50.00% and 83.33%, respectively.

**Conclusion:**

This study proposed that immunological changes assessed by PEA technology affect ovarian function in humans and that OSM may serve as a potential inflammatory biomarker for ovarian function in serum, thus revealing alterations in FF.

## Introduction

Many components in follicular fluid (FF), such as peptide hormones, cytokines, steroids, and energy metabolites, undergo dynamic changes during folliculogenesis, playing a positive or negative role in follicular development. In previous studies, researchers have shown that various immunology-related proteins, such as interleukins (IL-1 and IL-6) (1, 2), chemokines (CXCL8 and CCL11) (2, 3), vascular endothelial growth factor (VEGFA) (4), and extracellular matrix proteins (MMP7, ICAM3, and ITGA5) (5, 6), are involved in the process of folliculogenesis and follicle depletion. Furthermore, chronic low-grade systemic inflammation contributes to a diminished ovarian reserve (DOR) in mice (7), highlighting immunological changes in ovarian function. The latest research suggests that *EXOSC10* is essential for the maturation of oocytes, and blocking EXOSC10 activity can cause the rapid depletion of oocytes, disrupting ovarian reserve function and leading to a condition similar to primary ovarian insufficiency (POI) (8). We believe that the function of specific genes/proteins in the organism is not limited to one physiological role. Proteins that have a role in both oocyte maturation and ovarian reserve function will be more valuable in subsequent clinical applications and translational research.

Although FF is the most important microenvironment for oocyte development, the components of FF are closely linked to follicular development. However, owing to their inaccessibility (such as surgical retrieval), the components of FF are difficult to promote as biomarkers more widely. Serum and plasma samples are more convenient to obtain than FF samples and have been widely used in the diagnosis, treatment, and screening of new drug targets for various diseases (9, 10). Schweigert et al. (11) revealed significant similarities between serum and FF proteins during *in vitro* fertilization (IVF), with inevitable differences caused by selective transport. Therefore, we believe that the combined analysis of serum and FF could indicate the state of the ovarian microenvironment more intuitively.

During folliculogenesis, follicles become more permeable, resulting in a progressive increase in the number of serum proteins that pass through the blood−follicle barrier. Researchers have reported that the levels of the serum proteins IL-1 receptor antagonist (IL-1RA) (12) and retinol-binding protein-4 (RBP-4) (13) change during the menstrual cycle. Considering the correspondence with follicular development, we collected serum samples from the menstrual and ovulation phases for combined analysis.

Olink technology adopts a unique detection method referred to as the proximity extension assay (PEA), which combines traditional sandwich enzyme-linked immunosorbent assay (ELISA) with quantitative polymerase chain reaction (PCR)/second-generation sequencing (14), which increases the depth and sensitivity of proteome coverage. The Olink^®^ Target 96 Inflammation Panel contains 92 immunology-related proteins, including interleukins, chemokines, extracellular matrix proteins, and tumor necrosis factor. Nearly half of these proteins, such as IL-6, CXCL8, CCL11, VEGFA, AXIN1, SIRT2, and tumor necrosis factor superfamily member 12 (TWEAK), have been shown by previous researchers to be associated with oocyte development and maturation (2–4, 15–17). Moreover, the levels of inflammatory factors, such as IL-18 and TNF-α, can influence the ovarian reserve and embryo grade (18).

Therefore, we used the Olink^®^ Target 96 Inflammation Panel to analyze both FF samples and serum samples with the objective of determining the protein profile of oocyte maturation and the ovarian reserve. In addition to a bioinformatics analysis of differentially expressed proteins (DEPs), a Venn network of DEPs, and subsequent validation, novel inflammatory biomarkers for ovarian function were revealed. Special emphasis was placed on the Venn intersection of the DEPs because it may play a key role in ovarian function.

## Materials and methods

### Ethics statement

The complete details of the entire study design and procedures involved were in accordance with the Declaration of Helsinki. This study was approved by the Ethics Committee of the Faculty at Hangzhou Women’s Hospital (approval number: 2024-A-055, 2021-K7-02). Written informed consent was obtained from all the subjects before sample collection.

### Study design and sample collection

**Figure 1:**
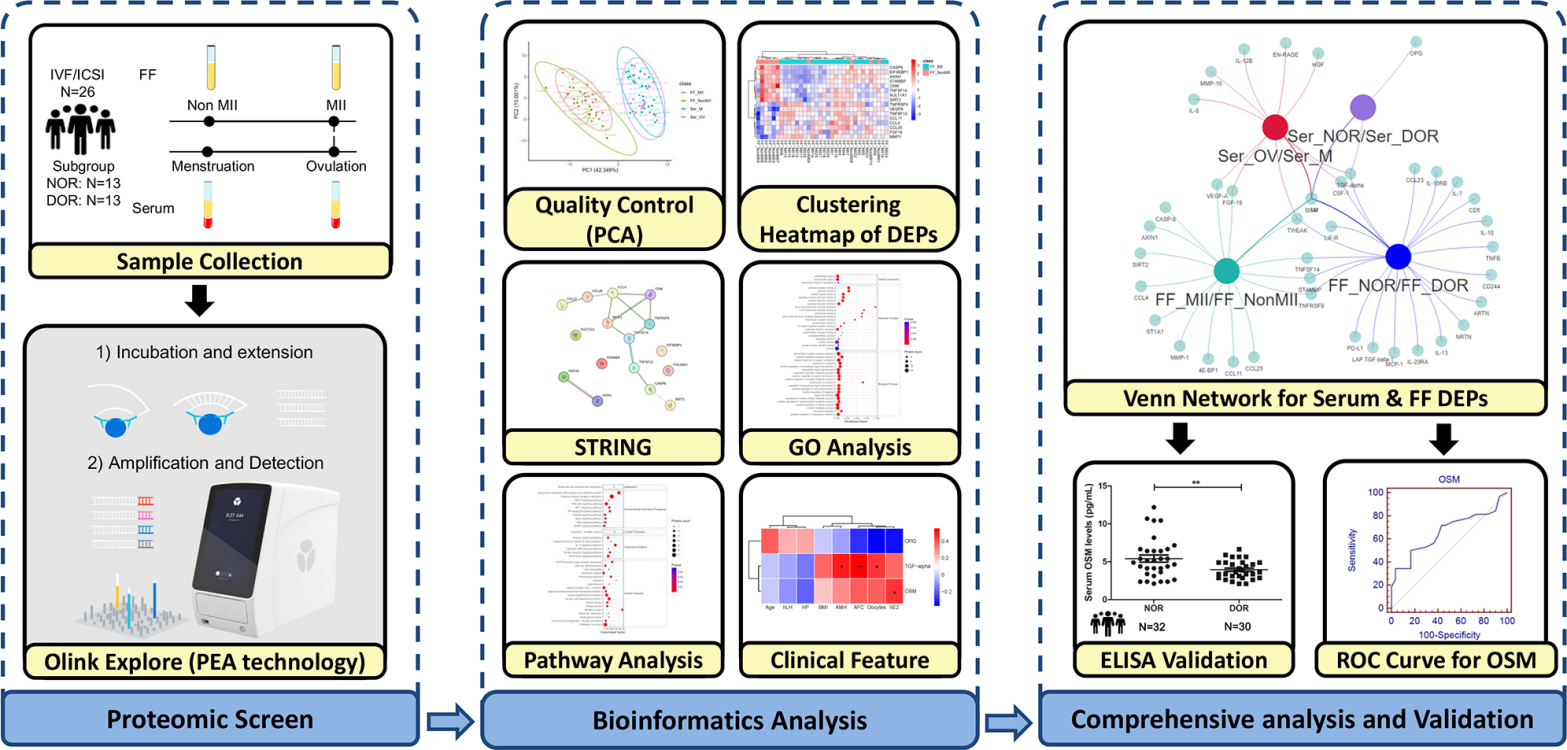
Workflow of the comprehensive analysis of inflammatory biomarkers of oocyte function. Olink PEA technology screening was applied to identify FF and serum protein changes during oocyte maturation, distinguishing subgroups through different ovarian reserves. After PCA analysis as quality control, we obtained the FF DEPs and serum DEPs. Bioinformatics analysis was performed for FF DEPs, including STRING analysis, GO term enrichment analyses, and pathway enrichment analyses. The correlations between the serum DEPs and clinical features were calculated. A Venn network was constructed among FF and serum DEPs, including different oocyte maturities and ovarian reserves. The OSM was validated via ELISA and evaluated via an ROC curve because it is the intersection of the Venn network. FF, follicular fluid; NOR, normal ovarian reserve; DOR, diminished ovarian reserve; PCA, principal component analysis; DEPs, differentially expressed proteins; GO, Gene Ontology; ROC, receiver operating characteristic.

We recruited a total of 88 subjects who underwent IVF, including intracytoplasmic sperm injection (ICSI), between January 2022 and May 2022 at Hangzhou Women’s Hospital. According to the Poseidon criteria (19), 26 subjects for Olink PEA proteomic technology were divided into a normal ovarian reserve group (NOR, N=13) and a DOR group (N=13). Furthermore, 62 subjects for ELISA validation were divided into 32 NOR samples and 30 DOR samples. In particular, DOR patients were defined as those with an antral follicle count (AFC) <5 and anti-Müllerian hormone (AMH) <1.2 ng/mL, and patients with an unexpected poor ovarian response (retrieving fewer than four oocytes). Patients who had undergone ovarian surgery or radiotherapy/chemotherapy, who had received steroid hormone therapy in the past 3 months, or who had a history of pelvic tuberculosis were excluded. The entire workflow is depicted in **Figure 1**.

Peripheral blood was collected from the participants during the menstruation and ovulation phases of the oocyte retrieval cycle. The menstruation phase was from the 2nd day of menstruation to the 5th day of menstruation, before the start of ovarian stimulation. The ovulation phase was determined by ultrasound monitoring of ovarian follicles and luteinizing hormone (LH) and estradiol (E2) measurements at the day of hCG administration. Blood for serum was collected, centrifuged at 3,000 × g for 10 min, and stored at -80°C.

Ovarian FF was collected from each follicle separately and allocated according to the maturity of the degranulated oocytes. According to the degranulation status of the oocytes, all 26 patients had mature oocytes (MII oocytes), whereas only 10 patients had both immature oocytes (MI and Gv oocytes) and mature oocytes. Immediately following oocyte retrieval, FF was collected and then centrifuged at 3,000 × g for 15 min to collect the supernatant. The supernatants were frozen at -80°C and stored.

We collected four samples from the same subjects for Olink PEA proteomic technology, including the serum from the menstruation phase (Ser_M), the serum from the ovulation phase (Ser_OV), the FF from mature oocytes (FF_MII), and the FF from immature oocytes (FF_NonMII). However, 16 subjects did not contain immature oocytes (FF_NonMII). Overall, we obtained 88 samples from 26 subjects for Olink exploration (26 Ser_M, 26 Ser_OV, 26 FF_MII, and 10 FF_NonMII) and DEPs between Ser_M vs. Ser_OV and FF_MII vs. FF_NonMII were analyzed. Furthermore, the Ser_OV group was divided into two subgroups, Ser_NOR (N=13) and Ser_DOR (N=13), and the FF_MII group was divided into two subgroups, FF_NOR (N=13) and FF_DOR (N=13), according to ovarian reserve parameters. DEPs between Ser_NOR vs. Ser_DOR and FF_NOR vs. FF_DOR were also analyzed. The serum of the ovulation phase for the ELISA validation subjects was also obtained (32 NOR samples and 30 DOR samples).

### Bioinformatics analysis of DEPs

In total, 92 protein names and UniProt IDs of the Olink^®^ Target 96 Inflammation Panel (Uppsala, Sweden) are shown in **Supplementary Table 1**. The samples are divided into four groups on the basis of their sources: Ser_M, Ser_OV, FF_MII, and FF_NonMII. Protein data were generated from serum and FF samples submitted to the Olink^®^ target 96 Inflammation Panel via PEA technology (20, 21). Normalized protein expression (NPX) values were provided as the final assay readout, which was an arbitrary log2-scale unit that corresponds to higher protein levels.

An NPX distribution box plot and a principal component analysis (PCA) plot were generated to check for outlier samples. After quality control of the original data (e.g., excluding control and outlier samples, merging and standardizing multiple sets of NPX data), clean data on NPX expression were obtained. The DEPs in the serum samples between the menstruation group and the ovulation group (Ser_OV/Ser_M) and in the FF samples between the mature group and the immature group (FF_MII/FF_NonMII) were compared. DEPs were also compared across subgroups (Ser_NOR/Ser_DOR, FF_NOR/FF_DOR) to identify biomarkers for ovarian reserve. Moreover, volcano plots were generated to visualize the DEPs (R software package “ggplot2”, V.3.3.5), and a clustering heatmap of the DEPs was also constructed (R software package “pheatmap”, V.1.0.12).

Using the STRING (http://www.string-db.org/) database, we conducted an interaction network analysis of the DEPs (encoding genes). All the FF DEPs were functionally classified (biological process, cellular composition, and molecular function) according to Gene Ontology analysis (GO, http://www.geneontology.org). Kyoto Encyclopedia of Genes and Genomes (KEGG, http://www.kegg.jp/) pathway analysis and enrichment analysis were performed via the R software package “ggplot2”, in addition to WikiPathways enrichment analysis (https://www.wikipathways.org/index.php/WikiPathways).

### Comprehensive analysis of serum and FF DEPs

The Pearson correlation method was used to compare the serum samples of the ovulation phase (Ser_OV) and the FF samples of mature oocytes (FF_MII). The proteins related to Ser_OV and FF_MII are listed.

The serum DEPs between the menstruation phase and the ovulation phase (Ser_OV/Ser_M) were defined as Group 1, and the FF DEPs between the mature oocyte and the immature oocyte (FF_MII/FF_NonMII) were defined as Group 2. The serum DEPs between different ovarian reserve cases (Ser_NOR/Ser_DOR) were defined as Group 3. The FF DEPs between different ovarian reserve cases (FF_NOR/FF_DOR) were defined as Group 4. A Venn network was constructed to assess the intersection among the four groups via Wekemo Bioincloud (https://www.bioincloud.tech) (22).

### Biomarker validation by ELISA methods

A human OSM ELISA kit (ab215543; Abcam, Cambridge, MA, USA) was used to assess protein levels in the serum. The protein concentrations were measured according to the manufacturer’s instructions. Differential serum OSM levels were measured in 32 NOR patients and 30 DOR patients. A human GM-CSF ELISA kit (CSB-E04568h; CUSABIO Co., Wuhan, China) and a human TNF-α ELISA kit (CSB-E04740h; CUSABIO Co., Wuhan, China) were used to assess protein levels in the FF.

### Statistical analysis

The parametric data are presented as the means ± SDs and were analyzed via t-tests to compare the means of two groups. The non-parametric data are presented as the median ± IQR and were compared via the Mann−Whitney U test. The statistical analyses of the Olink results were performed via the R software package “Olink Analyse” (V.1.2.6). Moreover, the correlations between serum DEPs and clinical characteristics were determined via the package “pheatmap” (0.3<| r |≤0.5, weak correlation; 0.5<| r |≤0.8, moderate correlation; and | r |>0.8, strong correlation). *P* values <0.05 were considered to indicate statistical significance. Receiver operating characteristic (ROC) curves were calculated using MedCalc Software (Version 12.4.2.0, Belgium). The diagnostic score for NOR was 1, whereas that of DOR was 0. The study sample provided 80.56% power to identify significant differences at a statistical significance level of α=0.05 for the Olink results (N_Ser_M_=25, N_Ser_OV_=23, after sample exclusion by PCA quality control) and provided 83.64% power for the ELISA results (N_Ser_NOR_=32, N_Ser_DOR_=30).

## Results

### Overview of the study subjects

Olink inflammatory proteomic analysis for oocyte maturity and ovarian reserve was performed with serum samples from subjects in the menstruation and ovulation phases and FF samples from subjects with mature and immature oocytes, including NOR and DOR samples. A total of 88 samples from 26 subjects were included in this cohort (26 Ser_M, 26 Ser_OV, 26 FF_MII, and 10 FF_NonMII). According to the PCA quality control, 82 samples were included in further analysis (**Supplementary Figure 1**, 25 Ser_M, 23 Ser_OV, 24 FF_MII, and 10 FF_NonMII). To verify the objectivity of our results, ELISA experiments on a known oocyte maturation marker in FF were performed (**Supplementary Figures 2A, B**).

Moreover, the Ser_OV and FF_MII groups were divided into the Ser_NOR, Ser_DOR, FF_NOR, and FF_DOR subgroups according to ovarian reserve, and the clinical characteristics of the NOR and DOR patients for Olink technology are shown in **Table 1**.

**Table 1:** Clinical and laboratory characteristics of the patients included in the Olink group.

	IVF/ICSI patients	
	NOR (N=13)	DOR (N=13)
Age (years)^a^	31.62 ± 2.33	33.85 ± 4.20
BMI (kg/m^2^)^a^	21.67 ± 3.37	22.51 ± 1.88
AMH (ng/mL)^b^	2.75 ± 1.64	1.07 ± 1.51^**^
AFC^b^	12.00 ± 3.00	5.00 ± 6.00^**^
Stimulation protocols (%)^c^		
Long protocol	100.00 (13/13)	23.08 (3/13)^**^
PPOS protocol	0.00 (0/13)	53.84 (7/13)
Mild stimulation protocol	0.00 (0/13)	23.08 (3/13)
Number of retrieved oocytes^b^	9.00 ± 6.00	2.00 ± 3.00^***^
Number of matured oocytes^b^	5.00 ± 5.00	1.00 ± 3.00^**^
Rate of matured oocytes (%)^a^	62.43 ± 24.23	82.69 ± 37.34
Rate of fertilization (%)^b^	68.54 ± 22.45	82.69 ± 37.34
Basal hormone level		
FSH (IU/L)^a^	3.86 ± 1.20	10.36 ± 5.93^**^
LH (IU/L)^b^	1.68 ± 1.55	2.37 ± 3.93
E2 (pg/mL)^b^	15.00 ± 4.27	31.98 ± 17.92^*^
P (ng/mL)^b^	0.43 ± 0.51	0.65 ± 0.54
Hormone level on hCG day		
LH (IU/L)^b^	2.22 ± 1.59	4.12 ± 7.16^*^
E2 (pg/mL)^a^	2689.35 ± 1076.93	930.48 ± 698.72^***^
P (ng/mL)^b^	0.89 ± 1.02	4.04 ± 8.95

Parametric data are presented as the means ± SDs, whereas non-parametric data are presented as the medians ± IQRs. NOR, normal ovarian reserve; DOR, diminished ovarian reserve; BMI, body mass index; AMH, anti-Müllerian hormone; AFC, antral follicle count; PPOS, progestin-primed ovarian stimulation; FSH, follicle-stimulating hormone; LH, luteinizing hormone; E2, estradiol; P, progesterone. ^a^ *P*-value between two groups was determined via the t-test; ^b^ *P*-value between two groups was determined via the Mann−Whitney U test; ^c^ *P*-value between three groups was determined via the chi-square test; ^*^ *P* < 0.05; ^**^ *P* < 0.01; and ^***^ *P* < 0.0001.

### Potential inflammatory biomarkers for oocyte maturation

**Figure 2:**
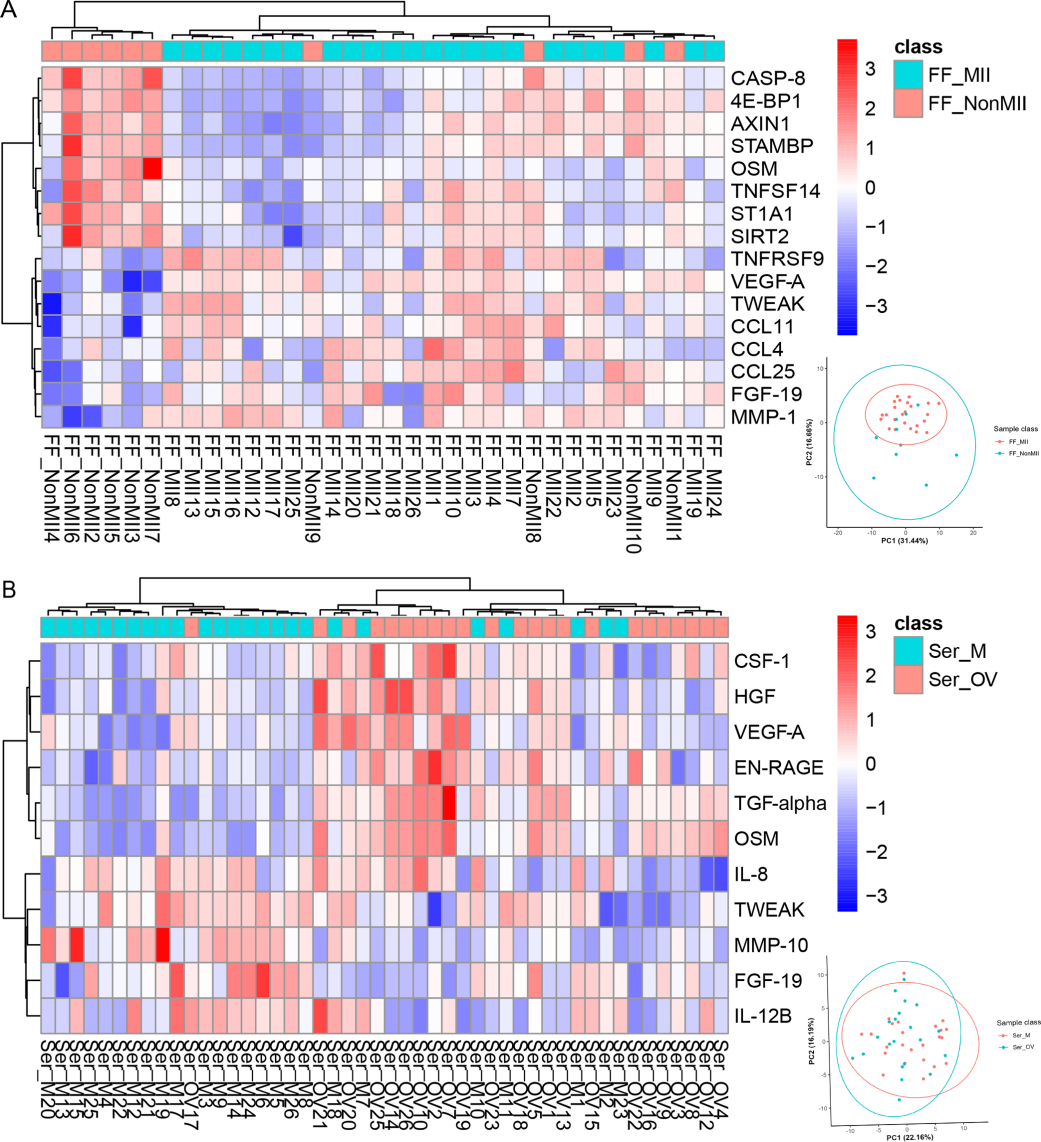
Differentially expressed proteins related to oocyte maturation identified via Olink PEA technology. Clustering heatmaps of **(A)** FF_MII vs. FF_NonMII and **(B)** Ser_OV vs. Ser_M were generated to visualize the expression patterns of the DEPs. The PCA results for each comparison group are shown in the lower right corner.

A linked proteomic analysis of FF from mature oocytes and FF from immature oocytes was performed to identify potential biomarkers of oocyte maturation. The expression of 16 proteins was significantly altered in the FF_MII group. TNFRSF9, VEGF-A, TWEAK, CCL11, CCL4, CCL25, FGF-19, and MMP-1 were upregulated in the FF_MII group, whereas CASP-8, 4E-BP1, AXIN1, STAMBP, OSM, TNFSF14, ST1A1, and SIRT2 were significantly downregulated in the FF_MII group. The heatmap shows clustering patterns of the DEPs between the two groups (**Figure 2A**).

We also screened the serum of the same patients at the ovulation and menstruation phases to identify potential serum/FF-correlated biomarkers for oocyte maturation. The expression levels of CSF-1, HGF, VEGF-A, EN-RAGE, TGF-α, and OSM were significantly elevated in the Ser_OV group. In contrast, the expression levels of IL-8, TWEAK, MMP-10, FGF-19, and IL-12B were significantly decreased in the Ser_OV group. A clustering heatmap of the serum samples is shown in **Figure 2B**.

### Potential inflammatory biomarkers for the ovarian reserve

**Figure 3:**
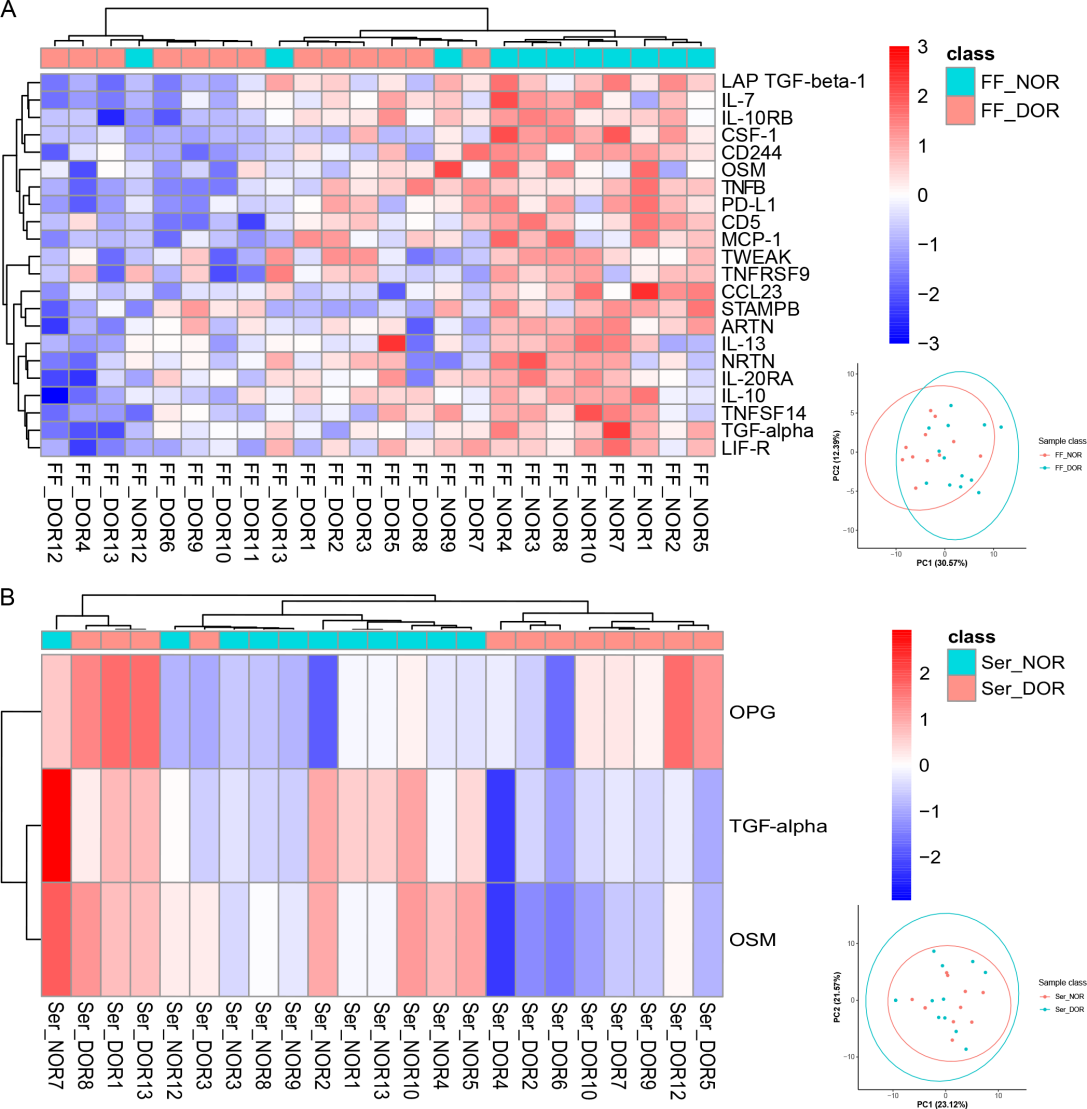
Differentially expressed proteins related to the ovarian reserve identified via Olink PEA technology. Clustering heatmaps of **(A)** FF_NOR vs. FF_DOR and **(B)** Ser_NOR vs. Ser_DOR were generated to visualize the expression patterns of the DEPs. The PCA results for each comparison group are shown in the lower right corner.

To identify potential biomarkers for ovarian reserve, we divided the FF_MII subgroup into the FF_NOR (N=11) and FF_DOR (N=13) subgroups and the Ser_OV subgroup into the Ser_NOR (N=11) and Ser_DOR (N=12) subgroups according to the Poseidon criteria. In total, 22 proteins—LAP TGF-β1, IL-7, IL-10RB, CSF-1, CD244, OSM, TNFB, PD-L1, CD5, MCP-1, TWEAK, TNFRSF9, CCL23, STAMBP, ARTN, IL-13, NRTN, IL-20RA, IL-10, TNFSF14, TGF-α, and LIF-R—were significantly upregulated in the FF_NOR group (**Figure 3A**). Only two upregulated proteins, namely, TGF-α and OSM, and one downregulated protein, OPG, were significantly altered in the Ser_NOR group (**Figure 3B**). The number of DEPs for each comparison and their corresponding *P* values are presented in **Supplementary Table 2**.

### Bioinformatics analysis of FF biomarkers for oocyte maturation and the ovarian reserve

**Figure 4:**
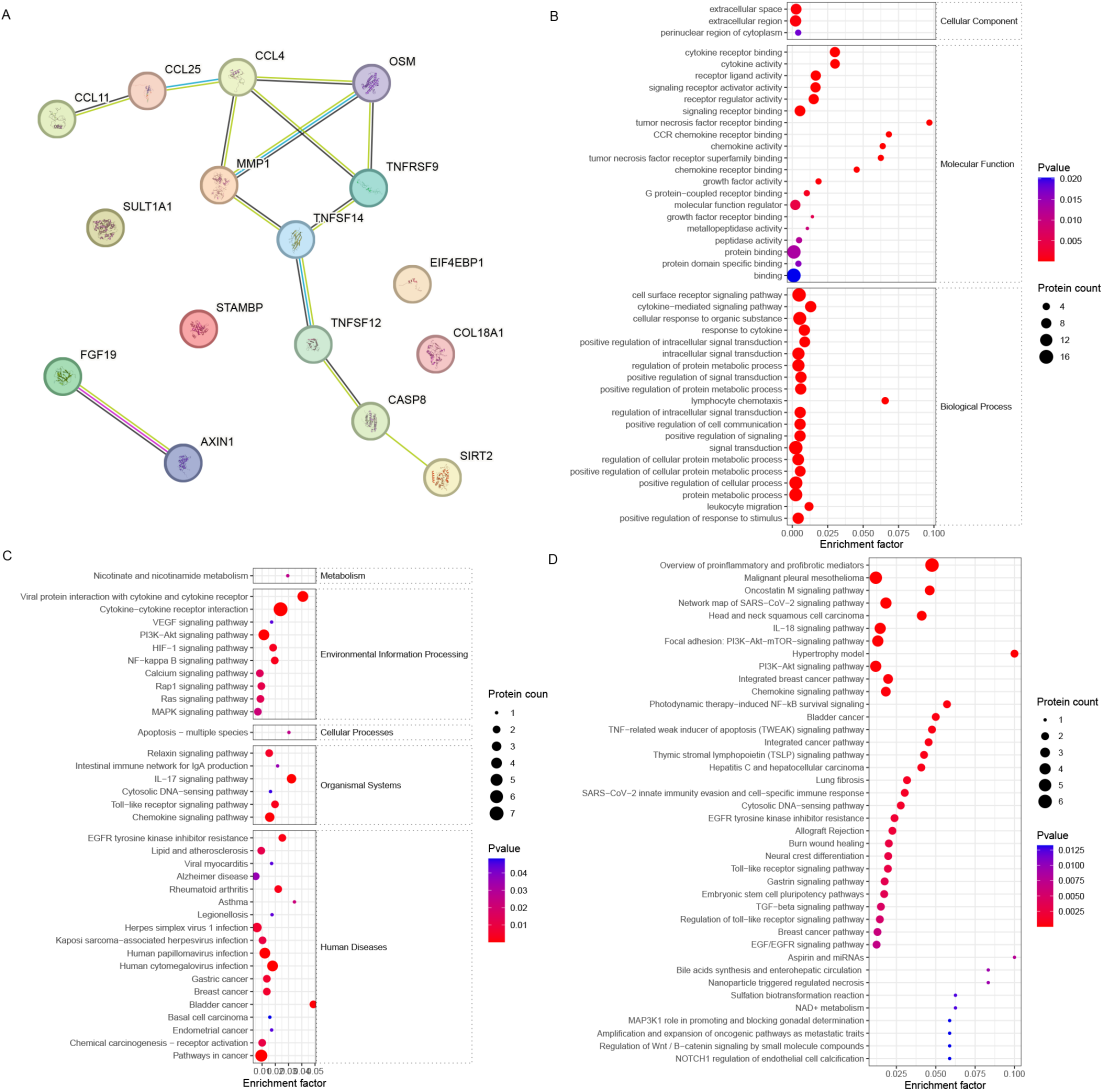
Bioinformatics analysis of the FF biomarkers for oocyte maturation. **(A)** STRING analysis revealed that the FF DEPs associated with different stages of oocyte maturity formed a good interaction network. **(B)** GO, **(C)** KEGG, and **(D)** WikiPathways enrichment analyses revealed different enriched terms for the DEPs.

The STRING analysis revealed interactions among DEPs (encoding genes) involved in oocyte maturation (**Figure 4A**). The DEPs related to oocyte maturation (FF_MII/FF_NonMII) were enriched in GO terms such as the cytokine-mediated signaling pathway (10 proteins), lymphocyte chemotaxis (four proteins), and leukocyte migration (six proteins) (**Figure 4B**). KEGG pathways such as the PI3K−Akt signaling pathway (four proteins), the NF−kappa B signaling pathway (two proteins), and the IL−17 signaling pathway (three proteins) were significantly enriched (**Figure 4C**). In addition, WikiPathways enrichment analysis highlighted the roles of proinflammatory and profibrotic mediators (six proteins), the oncostatin M signaling pathway (three proteins), the IL-18 signaling pathway (four proteins), and the PI3K-Akt-mTOR signaling pathway (four proteins) in oocyte maturation (**Figure 4D**).

**Figure 5:**
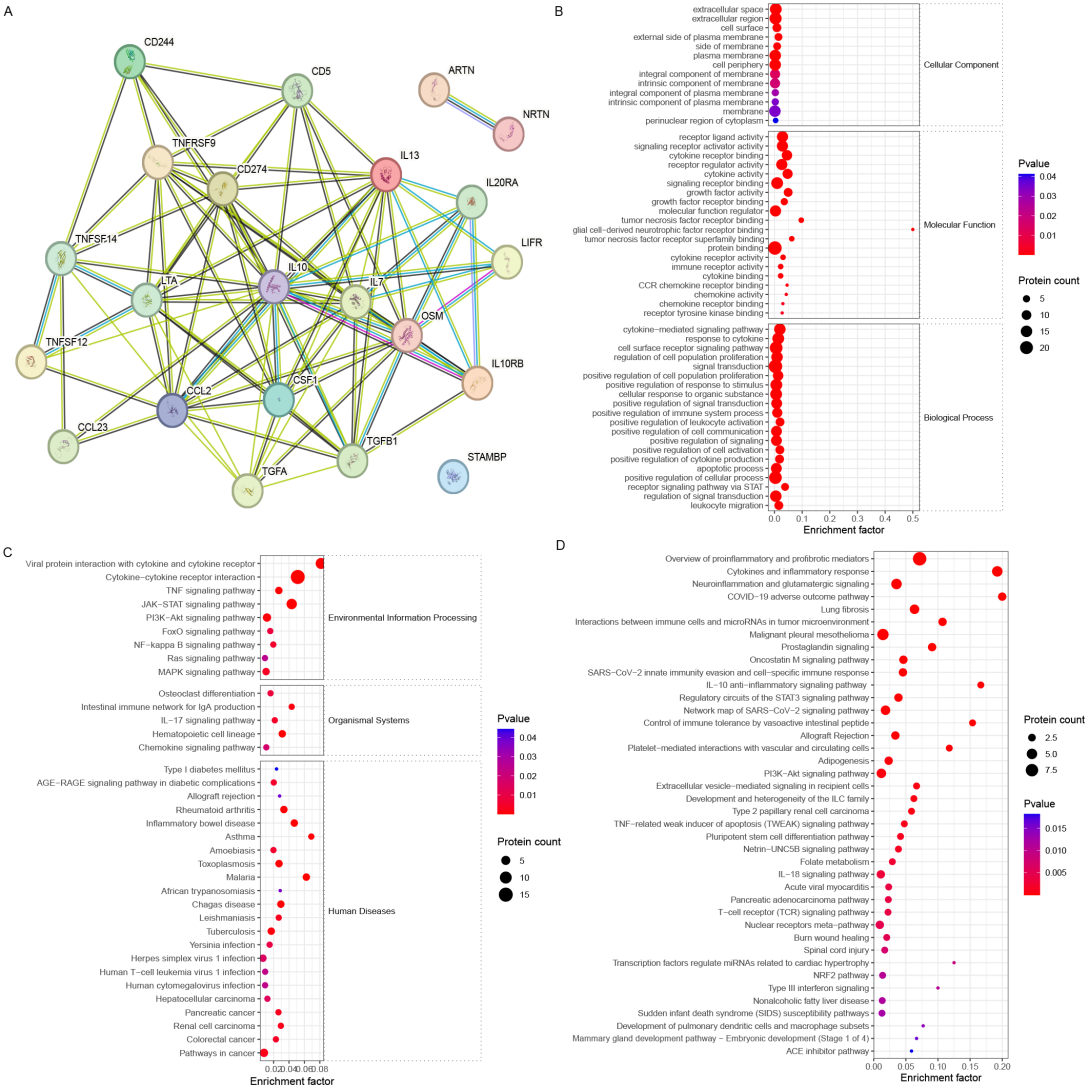
Bioinformatics analysis of the FF biomarkers for ovarian reserve. **(A)** STRING analysis revealed that FF DEPs with different ovarian reserves formed a good interaction network around IL-10. **(B)** GO, **(C)** KEGG, and **(D)** WikiPathways enrichment analyses revealed different enriched terms for the DEPs.

Further analysis revealed that the DEPs were associated with IL10, which is related to the ovarian reserve (**Figure 5A**). The DEPs related to ovarian reserve (FF_NOR/FF_DOR) were enriched in the receptor signaling pathway via STAT (six proteins) and positive regulation of leukocyte activation (eight proteins) (**Figure 5B**). The JAK-STAT signaling pathway (seven proteins), the PI3K-Akt signaling pathway (four proteins), the intestinal immune network for IgA production (two proteins), and the hematopoietic cell lineage (three proteins) were significantly enriched in the KEGG pathways (**Figure 5C**). Moreover, neuroinflammation and glutamatergic signaling (five proteins), the oncostatin M signaling pathway (three proteins), the IL-10 anti-inflammatory signaling pathway (two proteins), and the PI3K−Akt pathway (four proteins) were significantly enriched in the WikiPathways analysis (**Figure 5D**).

### Clinical correlation of serum biomarkers with the ovarian reserve

**Figure 6:**
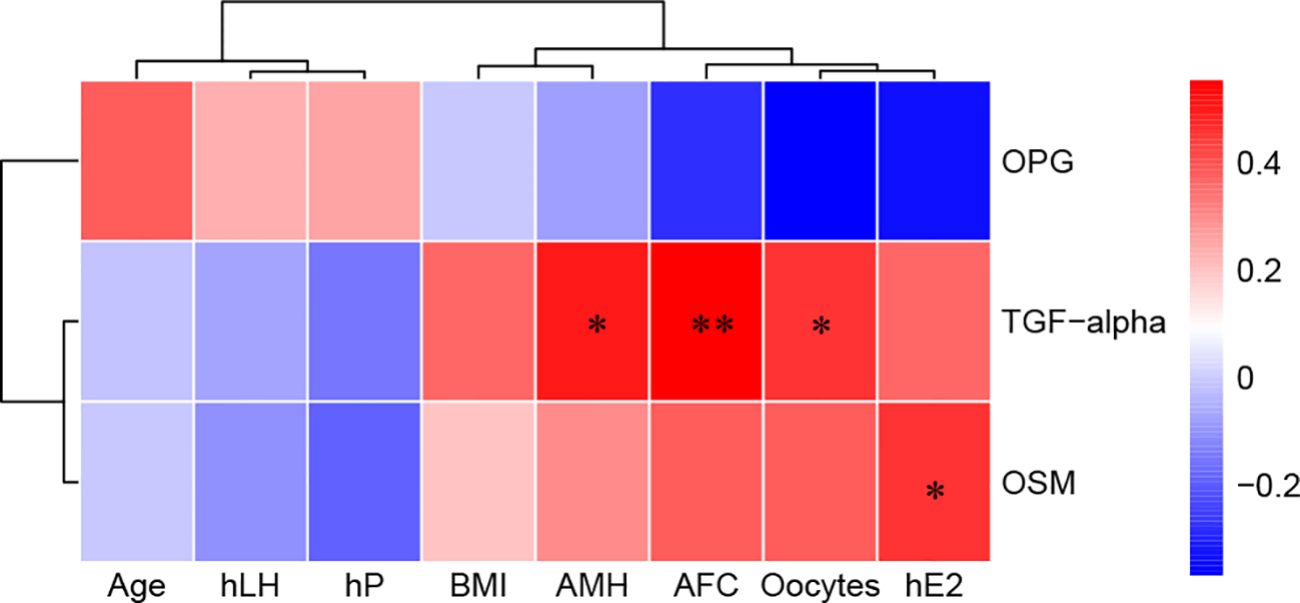
Correlations between serum biomarkers and clinical characteristics. Red, positively correlated; blue, negatively correlated, and white, not correlated. BMI, body mass index; AMH, anti-Müllerian hormone; AFC, antral follicle count; hLH, luteinizing hormone on hCG day; hE2, estradiol on hCG day; hP, progesterone on hCG day. ^*^ *P*< 0.05 and ^**^ *P*<0.01.

The associations between serum DEPs and different ovarian reserves (Ser_NOR vs. Ser_DOR group; ovulation phase; and TGF-α, OSM, and OPG) and clinical characteristics were analyzed. There were moderate correlations between TGF-α and AMH (*r*=0.503, *P*=0.017) and between TGF-α and AFC (*r*=0.552, *P*=0.006) and weak correlations between TGF-α and the number of retrieved oocytes (*r*=0.454, *P*=0.030), OSM and E2 on the hCG day (*r*=0.457, *P*=0.029) (**Figure 6**).

### Comprehensive analysis of inflammatory biomarkers for ovarian function

We investigated the correlation between the DEP concentrations in the serum samples of the ovulation phase (Ser_OV) and the FF samples of mature oocytes (FF_MII), and the results are shown in **Supplementary Table 3**. The DEPs obtained by Olink PEA technology, namely, CASP-8, EN-RAGE, CCL23, OSM, CCL4, CSF1, CD5, TNFB,TGF-α, CCL25, and AXIN1 showed expression correlation between Ser_OV and FF_MII.

**Figure 7:**
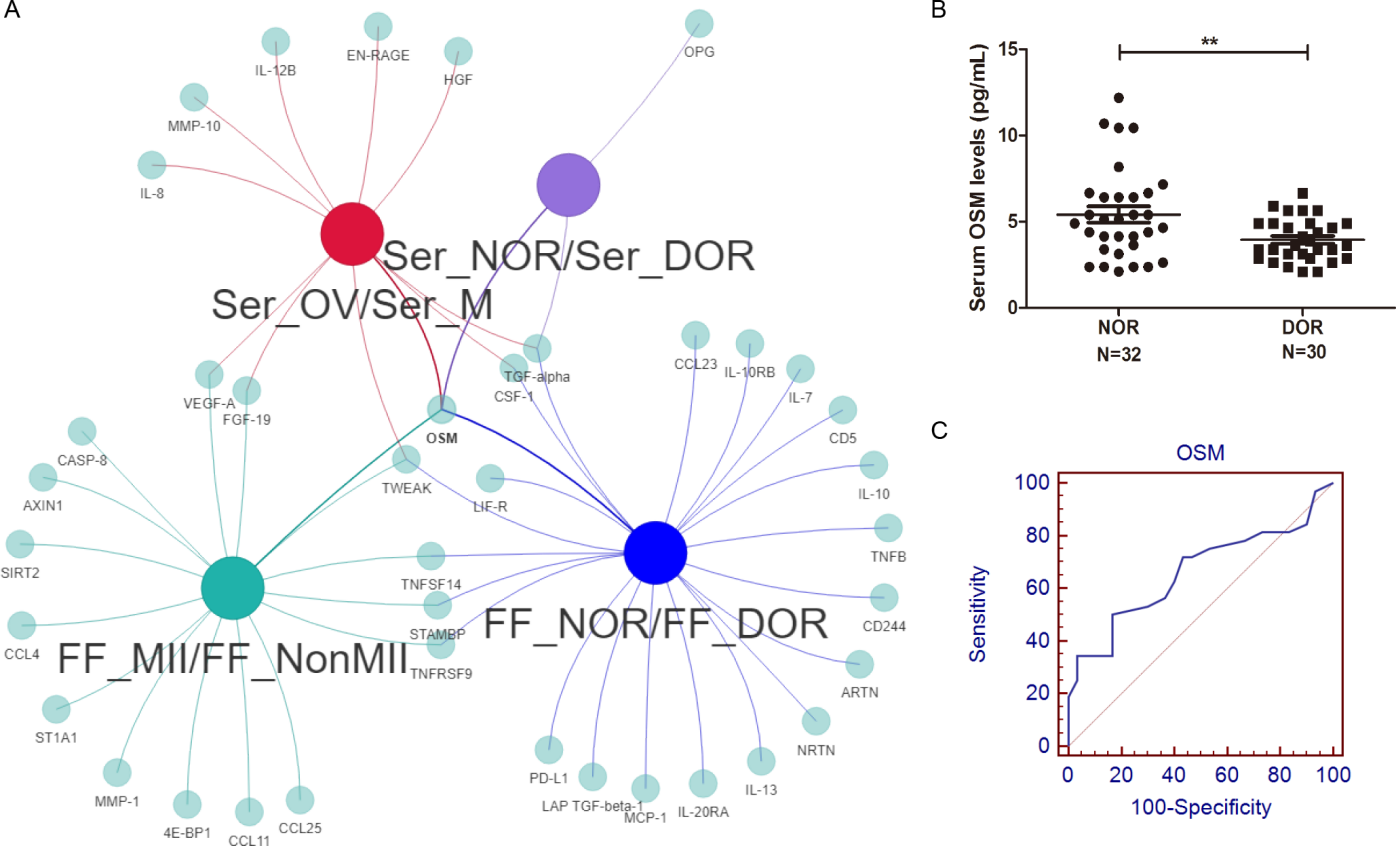
Comprehensive analysis and diagnostic value of the OSM. **(A)** A Venn network of FF and serum DEPs, including different oocyte maturities and ovarian reserves, was constructed. Among all four comparisons, only OSM showed consistently significant differences. **(B)** A significantly greater level of serum OSM was detected in NOR patients, and **(C)** the AUC of serum OSM for distinguishing different ovarian reserves was 0.661. ^**^ *P*<0.01.

We subsequently compared the serum DEPs and FF DEPs, which represent oocyte maturity and ovarian reserve, via a Venn network (**Figure 7A**). In the FF, five proteins, OSM, TWEAK, STAMPB, TNFRSF9, and TNFSF14, were altered at different stages of oocyte maturity and different stages of the ovarian reserve. Among the five proteins, only OSM was significantly altered in the serum.

### ELISA validation and diagnostic value of the OSM

To confirm our inflammatory proteomics results, we verified the serum OSM levels of NOR and DOR patients with a larger sample size for further clinical application. The clinical and laboratory characteristics are shown in **Table 2**. A significantly greater level of OSM (5.41 ± 2.65 vs.. 3.94 ± 1.23 pg/mL, *P*=0.007, **Figure 7B**) was detected in the NOR test. The level of OSM was associated with the AFC (r=0.351, *P*=0.005), the number of retrieved oocytes (r=0.345, *P*=0.006), the number of matured oocytes (r=0.430, *P*=0.001), and the basal hormone level of follicle-stimulating hormone (FSH) (r=-0.363, *P*=0.012). Meanwhile, the level of OSM was also associated with the level of GM-CSF (r=0.580, *P*=0.030, **Supplementary Figure 2C**), a known oocyte maturation marker. Moreover, we performed ROC analysis to evaluate the sensitivity and specificity of the OSM. The area under the curve (AUC) of serum OSM for distinguishing different ovarian reserves was 0.661 (0.530-0.776) (**Figure 7C**), the sensitivity was 50.00%, and the specificity was 83.33%.

**Table 2:** Clinical and laboratory characteristics of the patients included in the ELISA group.

	IVF/ICSI patients	
	NOR (N=32)	DOR (N=30)
Age (years)^a^	32.06 ± 4.27	37.17 ± 5.30^***^
BMI (kg/m^2^)^a^	22.57 ± 3.01	21.44 ± 2.98
AMH (ng/mL)^b^	2.92 ± 2.70	0.94 ± 0.87^***^
AFC^b^	10.00 ± 6.50	3.50 ± 2.25^***^
Stimulation protocols (%)^c^		
Long protocol	84.37 (27/32)	0 (0/30)^***^
PPOS protocol	9.38 (3/32)	80.00 (24/30)
Mild stimulation protocol	0.00 (0/32)	16.67 (5/30)
Antagonist	6.25 (2/32)	3.33 (1/30)
Number of retrieved oocytes^b^	9.00 ± 6.50	3.00 ± 2.00^***^
Number of matured oocytes^b^	8.00 ± 4.75	2.00 ± 2.00^***^
Rate of matured oocytes (%)^a^	80.16 ± 18.07	80.00 ± 29.41
Rate of fertilization (%)^b^	72.38 ± 21.07	71.28 ± 32.33
Basal hormone level		
FSH (IU/L)^b^	4.10 ± 2.69	8.52 ± 3.22^***^
LH (IU/L)^b^	1.85 ± 1.45	3.99 ± 2.94^***^
E2 (pg/mL)^b^	17.48 ± 13.04	32.84 ± 28.84^*^
P (ng/mL)^b^	0.54 ± 0.41	0.55 ± 2.26
Hormone level on hCG day		
LH (IU/L)^b^	2.63 ± 1.66	4.81 ± 4.89^**^
E2 (pg/mL)^b^	2563.00 ± 1717.00	837.20 ± 621.10^***^
P (ng/mL)^b^	0.76 ± 0.46	4.47 ± 5.79^***^

Parametric data are presented as the means ± SDs, whereas nonparametric data are presented as the medians ± IQRs. NOR, normal ovarian reserve; DOR, diminished ovarian reserve; BMI, body mass index; AMH, anti-Müllerian hormone; AFC, antral follicle count; PPOS, progestin-primed ovarian stimulation; FSH, follicle-stimulating hormone; LH, luteinizing hormone; E2, estradiol; P, progesterone. ^a^ *P*-value between two groups was determined via the t-test; ^b^ *P*-value between two groups was determined via the Mann−Whitney U test; ^c^ *P*-value between four groups was determined via the chi-square test; ^*^ *P* < 0.05; ^**^ *P* < 0.01; and ^***^ *P* < 0.0001.

## Discussion

Several proteomic studies have focused on alterations in FF proteins during follicular development and oocyte maturation (23, 24). Moreover, 2D PAGE analysis revealed that alpha-1-antitrypsin is the core protein that affects oocyte quality and ovulation in both serum and FF (25). However, data indicating that the serum protein concentration could also indicate oocyte maturity and ovarian function are lacking.

Our Olink PEA results revealed 16 proteins whose expression significantly differed between the FF of mature oocytes and the FF of immature oocytes: TNFRSF9, VEGF-A, TWEAK, CCL11, CCL4, CCL25, FGF-19, MMP-1, CASP-8, 4E-BP1, AXIN1, STAMBP, OSM, TNFSF14, ST1A1, and SIRT2 (**Figure 2**). Among them, CCL11 (3) in FF has already been shown to be altered during oocyte maturation in non-human primates, and VEGFA (4) in *in vitro* maturation (IVM) culture medium has been shown to improve the maturation of goat oocytes. Additionally, 4E-BP1 (26) and AXIN1 (15) have been shown to promote spindle assembly and cell cycle progression during meiotic maturation in mouse oocytes, whereas SIRT2 (16) has been shown to affect ovarian granulosa cell mitochondrial function during sheep oocyte maturation, as confirmed by molecular experiments. Owing to the high sensitivity of Olink technology, compared with our previous proteomic results, we detected many novel proteins, including AXIN1 and SIRT2, in FF (24).

Although PEA technology increases the depth and sensitivity of proteome coverage, it cannot reflect changes in the entire proteome, because it only analyzes specific targets. Therefore, Olink PEA technology is more suitable for combined analysis with other technologies covering the whole proteome or as a more in-depth study in a specific direction after proteomics screening.

FF is derived mainly from plasma. Considering the routine examination of serum sex hormones during controlled ovarian stimulation (COS), we also measured protein alterations during COS, mainly during the menstruation and ovulation phases, to identify biomarkers of oocyte maturation in the serum. Cytokines, such as VEGF-A, FGF-19, TWEAK, and OSM, which account for more of the FF DEPs, were also altered in the peripheral circulation (**Figure 7A**, intersection of the green and red nodes). Transcriptomic studies of mammalian cumulus−oocyte complexes (COCs) have confirmed the role of the VEGFA gene in IVM (27, 28), and supplementation with VEGFA has been shown to promote oocyte maturation and developmental potential (29, 30). Inconsistent with our results, no variation in the serum VEGF concentration was detected during the menstrual cycle in Zolton et al.’s study (31). FGF-19 may serve as an intermediary, resulting in typically reduced LH during the menopausal transition (32), whereas TWEAK suppressed the ovarian progesterone (P) content in gonadotropin-primed rats (17), which is related to sex hormones during the menstrual cycle.

In our study group, a long protocol, progestin-primed ovarian stimulation (PPOS) protocol, mild stimulation protocol, and an antagonist were used as stimulation protocols (**Tables 1**, **2**). PPOS and the mild stimulation protocol are commonly used in DOR patients with poor reserve ovarian parameters (AMH < 1.2 ng/mL, AFC < 5). The long protocol and antagonists are commonly used in populations with normal ovarian reserve parameters in our center. However, there are patients with sufficient prestimulation ovarian reserve parameters but unexpected poor ovarian responses in clinical practice. Meanwhile, we noticed that the P level of DOR patients on hCG day was significantly higher than that of NOR patients (**Tables 1**, **2**). Due to the DOR patients tending to use the PPOS protocol as their ovarian stimulation protocol, it was expected that the P level of this group would be elevated as they take two tablets of utrogestan (progesterone capsules) orally every day.

OSM is a cytokine of the IL-6 family, and with its receptors, OSM has been shown to be expressed in both oocytes and granulosa cells (33). Many studies have shown that OSM expression is closely related to ovarian and even reproductive system functions. In patients with polycystic ovary syndrome (PCOS), OSM is associated with obesity, insulin resistance, hyperandrogenism, and inflammation, ultimately resulting in androgen excess and ovulatory irregularities (34). OSM has also been found to be an important cause of ovarian cancer, cervical cancer, breast cancer, testicular cancer, and other cancers (35). In FF, a decreased level of OSM has been shown to negatively affect oocyte maturation (36), and the addition of OSM to IVM medium has been shown to rescue the maturation rate (37). However, previous studies have not addressed the expression level of OSM in the serum of IVF patients. In our study, the FF OSM level between mature oocytes and immature oocytes (**Figure 2A**), the FF OSM level between NOR patients and DOR patients (**Figure 3A**), the serum OSM level during the menstrual cycle (**Figure 2B**), and the serum OSM level between NOR patients and DOR patients (**Figures 3B**, **7B**) were significantly different. Moreover, serum OSM levels were weakly correlated with the number of matured oocytes (r=0.430, *P*=0.001) in ELISA validation, confirming our Olink results (**Figure 2A**). It was also correlated with a known oocyte maturation marker (r=0.580, *P*=0.030, **Supplementary Figure 2C**). Meanwhile, a weak correlation was found between FF and serum OSM (r=0.391, *P*=0.041; **Supplementary Table 3**). Tian et al. (38) noted that OSM is a risk factor for the onset of PCOS, which confirms that OSM may potentially improve ovarian responsiveness and the ovarian reserve. Therefore, we perceived that OSM is a potential inflammatory biomarker in both the peripheral circulating and ovarian microenvironments, representing oocyte maturity and the ovarian reserve. Additional studies are needed to clarify the molecular mechanisms through which OSM restores ovarian function via communication between the peripheral circulation and FF.

OSM can bind to two different receptors, leukemia inhibitory factor receptor (LIFR) and oncostatin M receptor (OSMR) (39). LIF-R and OSM were downregulated in DOR patients (**Figure 3A**). Therefore, we believe that the role of the OSM signaling pathway in ovarian function needs further exploration. Interestingly, FF inflammatory biomarkers for ovarian reserve are centered on IL-10 (**Figure 5A**), such as IL-13, CCL2, and TGF-β1. The association between the gene polymorphism of IL-13 and various infertility risk factors, such as PCOS, endometriosis, premature ovarian failure (POI), and DOR, has been confirmed (40). Genomic methods such as whole-exome sequencing and Illumina HiSeq technology have also confirmed the differential expression of the CCL2 (41) and TGF-β (42) signaling pathways in granulosa cells between DOR patients and NOR subjects. Wang et al. (43) demonstrated that decreased levels of IL-10 are linked to an elevated risk of POI via a bidirectional Mendelian randomization study. Our bioinformatics analysis also highlighted the role of the IL-10 anti-inflammatory signaling pathway in different ovarian reserves (**Figure 5D**). Correlation analysis revealed correlations between TGF-α and the following ovarian reserve indicators: AMH (*r*=0.503, *P*=0.017), AFC (*r*=0.552, *P*=0.006), and the number of retrieved oocytes (*r*=0.454, *P*=0.030) (**Figure 6**). Both TGF-α and LAP TGF-β1 were downregulated in DOR patients. Mito et al. (44) demonstrated that FSH and TGF-α synergistically increase porcine oocyte maturation via cumulus cells. Moreover, overexpression of TGF-α might result in reduced ovarian reserve and premature ovarian insufficiency in mice (45). Other studies have demonstrated that the TGF-β1/Smad3 signaling pathway contributes to the restoration of ovarian function in POI rats (46, 47). Therefore, we assumed that the OSM signaling pathway, the IL-10 anti-inflammatory signaling pathway, and the TGF signaling pathway are important inflammatory pathways for ovarian reserve capacity.

Our study investigated the changes in the inflammatory proteome affecting oocyte function in human FF and serum. Notably, OSM is a novel inflammatory biomarker in both the peripheral circulatory microenvironment and ovarian microenvironment and may be a potential therapeutic target for improving fertility, especially during ovarian ageing.

## Data Availability

The original contributions presented in the study are included in the article/**Supplementary Material**. Further inquiries can be directed to the corresponding authors.

## Supplementary material

Supplementary Figure 1 PCA quality control results of the Olink investigation.

Supplementary Figure 2 ELISA results of known oocyte maturation markers in FF and their correlation with OSM.

Supplementary Table 1 The protein list of Olink^®^ Target 96 Inflammation Panel.

Supplementary Table 2 Number of DEPs for each comparison and their corresponding *P*-values.

Supplementary Table 3 Correlation between the serum of ovulation phase and FF by Olink exploration.
