# Supplementary figures and images for: Proximity extension assay revealed novel inflammatory biomarkers for follicular development and ovarian function: a prospective controlled study combining serum and follicular fluid
Source: Front Endocrinol (Lausanne). 2025 Feb 10;16:1525392. doi: 10.3389/fendo.2025.1525392 (PMC11847672; doi:10.3389/fendo.2025.1525392)

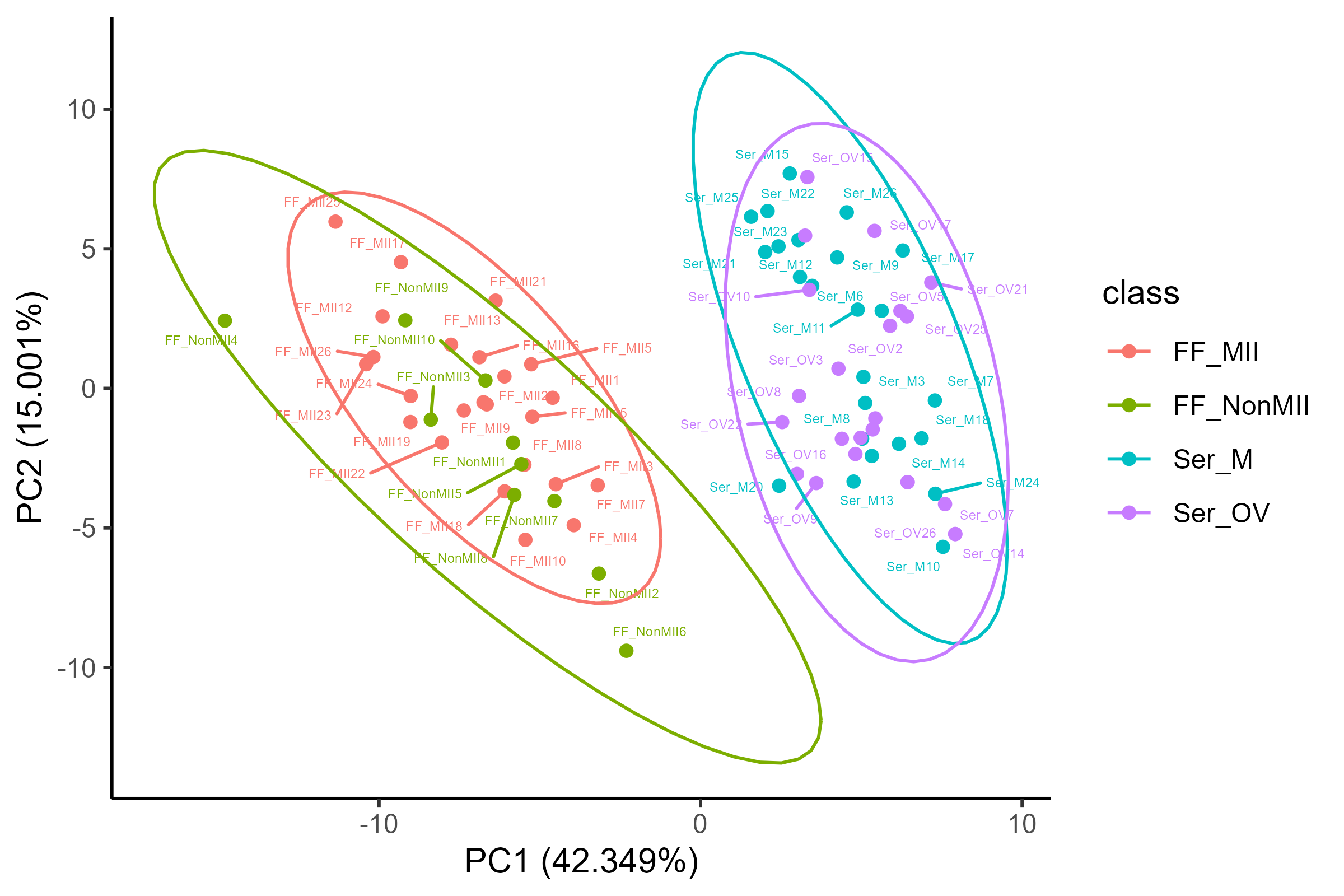

Supplement: Supplementary Figure 1 — PCA quality control results of the Olink investigation. [file Image1.tif]

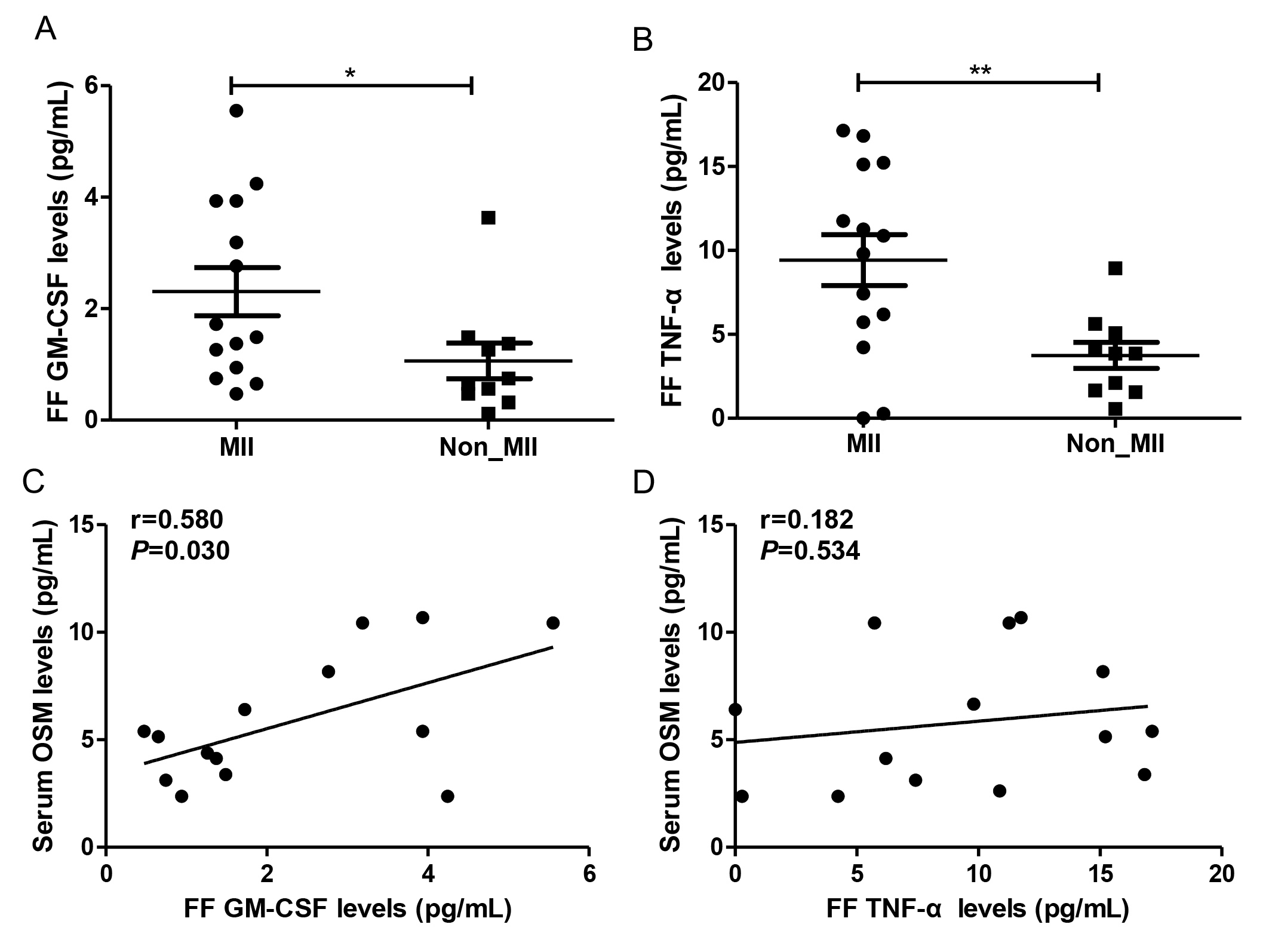

Supplement: Supplementary Figure 2 — ELISA results of known oocyte maturation markers in FF and their correlation with OSM. [file Image2.tif]
