# Supplementary material for: Proximity extension assay revealed novel inflammatory biomarkers for follicular development and ovarian function: a prospective controlled study combining serum and follicular fluid
Source: Front Endocrinol (Lausanne). 2025 Feb 10;16:1525392. doi: 10.3389/fendo.2025.1525392 (PMC11847672; doi:10.3389/fendo.2025.1525392)
Supplement: Supplementary Table 3 — Correlation between the serum of ovulation phase and FF by Olink exploration. [file Table3.doc]

Table S3 Correlation between the serum of ovulation phase and FF by Olink exploration.

| Protein symbol | Uniprot ID | r | *P* value |
| --- | --- | --- | --- |
| Positive correlation | |  |  |
| IL-10RA | Q13651 | 0.871 | < 0.0001 |
| CCL19 | Q99731 | 0.859 | < 0.0001 |
| IL18 | Q14116 | 0.843 | < 0.0001 |
| CD8A | P01732 | 0.818 | < 0.0001 |
| FGF-21 | Q9NSA1 | 0.775 | < 0.0001 |
| IL-12B | P29460 | 0.761 | < 0.0001 |
| CCL25 | O15444 | 0.745 | < 0.0001 |
| SCF | P21583 | 0.738 | < 0.0001 |
| IL-10RB | Q08334 | 0.717 | < 0.0001 |
| MCP-2 | P80075 | 0.672 | < 0.0001 |
| CD6 | P30203 | 0.609 | 0.0002 |
| Flt3L | P49771 | 0.609 | 0.0002 |
| TNF | P01375 | 0.594 | 0.0003 |
| IL-18R1 | Q13478 | 0.589 | 0.0003 |
| TRAIL | P50591 | 0.564 | 0.0008 |
| ADA | P00813 | 0.541 | 0.0017 |
| DNER | Q8NFT8 | 0.520 | 0.0029 |
| TGF-alpha | P01135 | 0.518 | 0.0031 |
| CCL23 | P55773 | 0.499 | 0.0050 |
| uPA | P00749 | 0.461 | 0.0118 |
| CDCP1 | Q9H5V8 | 0.459 | 0.0122 |
| CD5 | P06127 | 0.439 | 0.0181 |
| EN-RAGE | P80511 | 0.433 | 0.0204 |
| CSF-1 | P09603 | 0.427 | 0.0228 |
| TNFB | P01374 | 0.426 | 0.0231 |
| CST5 | P28325 | 0.420 | 0.0257 |
| IL2 | P60568 | 0.398 | 0.0374 |
| IL-1 alpha | P01583 | 0.397 | 0.0375 |
| CD40 | P25942 | 0.397 | 0.0381 |
| CCL3 | P10147 | 0.396 | 0.0383 |
| IFN-gamma | P01579 | 0.395 | 0.0388 |
| OSM | P13725 | 0.391 | 0.0412 |
| AXIN1 | O15169 | 0.388 | 0.0434 |
| CASP-8 | Q14790 | 0.381 | 0.0484 |
| CCL4 | P13236 | 0.379 | 0.0497 |
| Negative correlation | |  |  |
| IL-17C | Q9P0M4 | -0.427 | 0.0424 |

The Pearson correlation method was performed to determine the association between two different parameters. 0.3<| r |≤0.5, weak correlation; 0.5<| r |≤0.8, moderate correlation; and | r |>0.8, strong correlation.
